# Psychological Well-Being of Trans* People in Italy During the COVID-19 Pandemic: Critical Issues and Personal Experiences

**DOI:** 10.1007/s13178-021-00633-3

**Published:** 2021-08-14

**Authors:** Marta Mirabella, Giulia Senofonte, Guido Giovanardi, Vittorio Lingiardi, Alexandro Fortunato, Francesco Lombardo, Anna Maria Speranza

**Affiliations:** 1 Department of Dynamic and Clinical Psychology, and Health Studies, Sapienza University of Rome, Via degli Apuli 1, 00185 Rome, Italy; 2 Laboratory of Seminology, Sperm Bank “Loredana Gandini,” Department of Experimental Medicine, Sapienza University of Rome, Rome, Italy

**Keywords:** COVID-19, Transgender, Gender identity, Access to care, Psychological well-being, Risk factors

## Abstract

**Introduction:**

The COVID-19 pandemic represents risk to physical health and psychological well-being. Specifically, it causes concerns among minoritized communities, such as transgender/non-binary individuals. The study investigates the experience of this group in Italy during the pandemic and identifies risk factors that exerted negative impacts on psychological well-being and physical health.

**Methods:**

The study developed an online questionnaire to investigate psychological status, access to medical health services, and factors such as predictors of risk outcomes among transgender/non-binary people during lockdown restrictions. Responses were collected (*n* = 256) on April 2020. Descriptive analysis, univariate analysis of variance, and *t*-test were conducted.

**Results:**

The respondents confirmed several issues, such as worries about the future (58.2%), negative emotions (46.9%), and self-uncertainty (52.7%) during the pandemic. Difficulties in undertaking hormone therapy and access to health facilities were reported. Living with family members represented a significant stressor for psychological well-being, whereas lack of support from LGBT + communities was reported. Subjects undergoing psychological therapy during the pandemic reported increases in depression, anxiety, somatization, and lack of privacy in the household.

**Conclusions:**

The study findings provide a framework for critical aspects experienced by transgender/non-binary individuals during the pandemic. Specifically, the study provides evidence of the vulnerability of this population underlined by multiple difficulties.

**Policy Implications:**

The study findings provide an overview of the experience of transgender and non-binary people during the pandemic, insights regarding risk factors, and aspects that require adequate attention and care.

**Supplementary Information:**

The online version contains supplementary material available at 10.1007/s13178-021-00633-3.

## Introduction

In March 2020, the World Health Organization (World Health Organization, 2020) declared the coronavirus disease epidemic of 2019 a worldwide pandemic due to its spread in various geographical areas. As of March 2021, Italy had more than 3.72 million confirmed cases and ranked second in terms of mortality among the European countries.

Apart from morbidities and mortality, previous studies provided evidence that any major epidemic exerts negative effects on individuals and society (Guan et al., 2020). In particular, such effects may likely entail disproportionate consequences and increase inequalities, particularly for individuals belonging to marginalized communities, such as transgender people (Perez-Brumer & Silva-Santisteban, 2020; Salerno et al., 2020). Indeed, given the significant difficulties and interpersonal challenges that transgender people constantly face (Bockting et al., 2016), they may have experienced a significant increase in these struggles with the implementation of strict quarantine measures.

In this respect, an editorial (van der Miesen et al., 2020) and several studies (Gava et al., 2021; Hawke et al., 2021; Perez-Brumer & Silva-Santisteban, 2020; Salerno et al., 2020; Wang et al., 2020) underlined the significant burden that the pandemic may represent for transgender people. First, transgender individuals were forced into unsupportive and potentially unsafe households as a result of social and physical distancing measures implemented to contain the spread of COVID-19. Such a situation may have exposed them to increased experiences in proximal minority stress (Salerno et al., 2020). In this respect, previous studies (Giovanardi et al., 2019; Grossman et al., 2005, 2019) provided evidence that households are seemingly a significant stressor for the psychological well-being of transgender individuals. This group frequently experiences unsupportive and unsafe households, which are characterized by aversive relationships with parents, severe rejection, neglect, and psychological abuse (Domínguez-Martínez et al., 2020; Giovanardi et al., 2018; Grossman et al., 2006; Peng et al., 2019; Salerno et al., 2020). Moreover, Perez-Brumer and Silva-Santisteban (2020) demonstrated that social restrictions in several countries, such as Peru, included limitations based on gender. Consequently, men and women were allowed to step outside and leave their homes only on certain weekdays (i.e., men: Wednesday and Friday; women: Tuesday, Thursday, and Saturday), which led to significant discrimination and stigma for transgender individuals.

Given these limitations, the lack of social relationships may also have impacted this group, in addition to the sense of loneliness and the lack of support from social relationships (van der Miesen et al., 2020). In fact, the absence of social relationships for transgender people represents a risk factor for their psychological well-being, thus leading to the development of high levels of distress, discrimination, isolation, and sense of loneliness (Johnson & Amella, 2014).

An editorial by van der Miesen et al. (2020) also underlined how priority in terms of health care during the pandemic may affect gender-affirming care, causing several deferrals and limitations, leading to increased risk of self-medication. Such insufficient access to gender-affirming care or deferrals in receiving care may add a significant mental burden on transgender people (van der Miesen et al., 2020) apart from their mental health issues, severe body discomfort, and risk of undergoing complex behavioral patterns, such as self-harm and eating disorders (Mirabella et al., 2020). In this regard, Wang et al. (2020) mentioned difficulty in accessing gender-affirming care and its association with high levels of depression and anxiety due to uncertainty about the future and availability of care.

Recently, Jarrett et al. (2020) and Koehler et al. (2020) reported the effects of the pandemic on transgender people and confirmed its significant impact on the psychological well-being of subjects who experienced deferrals in gender-affirming care. In Italy, care for transgender people is complex and multifaceted and, as suggested by the latest guidelines, promoted by the Standards of Care for transsexual, transgender, and gender-non-conforming people developed by the World Professional Association for Transgender Health (SOC 7, WPATH, 2011), should be focused on psychological and endocrinological support and family involvement, particularly for youths. Currently, several centers in Italy specialized in gender-affirming care (e.g., Turin, Bologna, Florence, and Rome) are subjected to national coordination with the National Observatory of Gender Incongruence (ONIG, 2017). These services, however, do not cover all Italian territories, and centers present individual characteristics that are dependent on local (regional) directives in terms of supply of medicine, specialized visits, and access to specific services. Therefore, the framework implicates difficulties of networking among centers and disparities for transgender people who live in certain areas of Italy and consequently must migrate to specialized centers. In an emergency, such as the COVID-19 pandemic that is pressing on the national health system and with lockdown measures that restrict movement, access to care for transgender people may result in further challenges, which may put the psychological well-being of transgender individuals at risk. Although various studies have addressed the impact of the COVID-19 pandemic on transgender individuals and have focused on the several possible burdens this population is facing, the dearth in studies has deepened, especially with regard to the impact of various issues on their psychological well-being.

For these reasons, the current study evaluates the impact of COVID-19 on the psychological well-being of the transgender and non-binary population throughout Italy. It investigates the effect of household conditions, limited social relationships, and difficult access to health care and services. It also provides an overview of the psychological support received by transgender and non-binary people during lockdown through their descriptions of personal experiences and critical issues. Furthermore, this study provides suggestions for the improvement of health and the organization of care for transgender people in Italy.

## Methods

### Instrument and Procedure

An online survey was created ad hoc for this study (see Supplementary Appendix). The development of the questionnaire was based on a multi-step process. First, items exploring the demographic features of the respondents, such as age, gender at birth, gender identity, level of education, civil status, job, and residence, were collected. Second, based on a review of the literature (Perez-Brumer & Silva-Santisteban, 2020; Salerno et al., 2020; van der Miesen et al., 2020; Wang et al., 2020), the study identified topic-specific items investigating relevant issues that transgender non-binary people may have faced during the pandemic, such as discrimination episodes in the household, difficulties in obtaining medical prescriptions, lack of access to medicines and non-pharmacological devices, access to health services, or delays in hormone administration and surgeries. Third, subscales investigating the psychological well-being of the respondents during the COVID-19 pandemic were added. Specifically, the study derived such subscales from the Symptom Checklist-90 (SCL-90) (Derogatis & Cleary, 1977), which is a 90-item multidimensional self-report symptom inventory, and from the Well-Being Questionnaire Short Form (W-BQ12; Riazi et al., 2006), which investigates the degree of the quality of well-being. Regarding the SCL-90 (Derogatis & Cleary, 1977), only the Depression, Anxiety, Hostility, and Somatization subscales were used. The Depression subscale reflects a range of depressive symptoms, such as feelings of hopelessness and helplessness, lack of motivation, low self-esteem, signs of withdrawal from life interest, and loss of vital energy. The Anxiety subscale investigates the general signs of anxiety, such as nervousness, tension, trembling, difficulty in concentrating, racing thoughts, feeling irritable and nervous, and several other somatic manifestations of anxiety. The Hostility subscale includes thoughts, feelings, and actions characteristic of irritability and anger. Finally, the Somatization subscale reflects distress emerging from perceptions of bodily dysfunction. Symptoms may pertain to the cardiovascular, gastrointestinal, respiratory, and neurological systems. With respect to the Well-Being Questionnaire Short Form, the study employed the positive well-being dimension. Fourth, items investigating the perceived availability of support from the LGBT + community during the pandemic and whether the respondents underwent psychological therapy before or during the COVID-19 pandemic were created. Moreover, items that elicited related experiences during the lockdown were added. Following these steps, a total of 118 questions (open-ended, Likert-type, and multiple-choice questions) were produced and grouped under five parts, namely, demographic aspects, gender identity and COVID-19, transition path and COVID-19, social media and communication, and psychological well-being during the pandemic. Google Forms were employed to conduct the survey, which was released online on April 25, 2020, and remained online for 3 weeks. The survey was advertised through social media platforms and web groups of Italian transgender people, gender clinics, and support groups, and via word-of-mouth.

The Ethics Committee of the Department of Developmental and Clinical Psychology, Sapienza University of Rome approved the study. The participants provided written informed consent in the first part of the questionnaire. Participation in the study was entirely voluntary and without financial compensation.

### Participants

The inclusion criteria were transgender/non-binary people, aged 18 years or older, and living in Italy. A total of 256 responses were collected (see Table 1).

**Table 1:** Demographic characteristics of the sample (*N* = 256)

**Variable**	***N* (%)**
*Sex assigned at birth*	
Male	61 (23.8)
Female	189 (73.8)
Missing	6 (2.4)
*Age (mean* ± *standard deviation*)	28.9 ± 10.7
* Gender identity*	
Male	11(4.3)
Woman	31 (12.1)
Transgender	29 (11.3)
Man	44 (17.2)
Masculine	34 (13.3)
Feminine	15 (5.9)
Boy FtM	18 (7.0)
Fluid	3 (1.2)
Nonbinary	18 (7.0)
Female	4 (1.6)
MtF	2 (0.8)
Incomplete	4 (1.6)
Demiboy	2 (0.8)
Binary	2 (0.8)
Secure	3 (1.2)
Complicated	3 (1.2)
Genderfluid	4 (1.6)
Boy	2 (0.8)
Agender	2 (0.8)
Easy	6 (2.3)
Undefined	3 (1.2)
Other	13 (5.1)
*Education level*	
Primary school	1 (0.4)
Middle school	58 (22.7)
High school	139 (54.3)
Bachelor’s degree	34 (13.3)
Master’s degree	16 (6.3)
Postgraduate	8 (3.1)
*Occupation*	
Student	97 (37.9)
Self-employed	30 (11.7)
Unemployed	55 (21.5)
Employed	70 (27.4)
Retired	3 (1.2)
Owner pension income	1 (0.4)
*Change of residence because of the Covid lockdown*	
No	211 (82.4)
Yes	26 (10.2)
I considered it, but I could not	19 (7.4)

### Data Analysis

The study conducted a descriptive analysis of the sample. Univariate analysis of variance (ANOVA), after conducting Levene’s correction for inequality of variance, was used to compare the continuous variables among the groups. Bonferroni’s post hoc test was performed for pairwise comparison among the groups. *t*-Test was used to compare dichotomous variables, and MANCOVA/GLM was employed to better assess differences between the groups of categorical independent variables on the dependent variables. Analyses were conducted with IBM SPSS Statistics, version 25 (IBM Corp., Armonk, NY, USA).

## Results

### Demographic Characteristics of the Sample

Table 1 reports the demographic characteristics of the sample. The majority of the participants were assigned female at birth (73.8%), and the mean age of the participants indicates that the majority is aged under 30 years. Regarding the gender identity of the respondents, the results indicated a numerous range of identities, which implied an open perception of oneself.

The majority of participants (37.9%) were students, a total of 26.6% were employed, whereas 21.5% were unemployed. In terms of educational level, the majority of the sample achieved a high school diploma (54.3%), whereas only 3.1% completed a postgraduate degree. As shown in Table 1, the majority of the participants (82.4%) retained the same residence during the COVID-19 pandemic, whereas most of them (36.7%) reported living in the suburbs of a large city in a three-room apartment (68.4%). Among these residences, 88.7% had a terrace, balcony, or garden, whereas only 11.3% lacked open spaces. Regarding housing conditions, 13.3% of the participants lived alone, whereas 86.7% lived with others, out of which 60.9% are family members, 14.8% are partners, 6.6% are roommates, and 4.3% are unspecified.

### Psychological Status

Regarding the psychological status of respondents during COVID-19, this study employed four subscales, namely, Depression, Hostility, Anxiety, and Somatization, from the SCL-90 (Derogatis & Cleary, 1977) and one subscale (Positive Emotions) from the Well-Being Questionnaire Short Form (W-BQ12; Riazi et al., 2006). Table 2 shows that the mean scores for Depression (17.83 ± 13.78) are higher than those for other scales, whereas the mean scores for the Positive Emotions scale (7.17 ± 4.63) were the lowest compared with those for the other subscales. After a detailed examination of the psychological items (Table 3), analysis of frequencies demonstrated that respondents reported feelings of discouragement about the future (18.8%) and cited being unable to be free from worries (58.2%), unable to feel positive emotions (46.9%), uncertain about oneself (52.7%), anxious (25.4%), and unable to remain relaxed and calm (39.8%). Moreover, investigating the differences perceived by respondents in terms of psychological well-being, 47.7% reported negative changes within the period before the COVID-19 pandemic, which confirms the impact of the pandemic on mental health. Specifically, 7.8% of the respondents reported positive changes in psychological status within the same period.

**Table 2:** Psychological scales

**Scale**	**Mean**	**Standard deviation**
**Hostility**	6.19	4.18
**Depression**	17.83	13.78
**Anxiety**	9.01	6.52
**Somatization**	11.25	6.80
**Positive Emotions**	7.17	4.63

**Table 3:** Psychological status of the sample (*N* = 256)

**Variable**	***N* (%)**				
	**Not at all**	**Slightly**	**Moderately**	**Very**	**Extremely**
I feel nervous	37 (14.5)	69 (27.0)	68 (26.6)	59 (23.3)	23 (9.0)
I feel anxious	46 (18.0)	59 (23.0)	65 (25.4)	59 (23.0)	27 (10.5)
I’m afraid of losing control	110 (43.0)	57 (22.3)	34 (13.3)	39 (15.2)	15 (5.9)
I do not know what is happening inside me	135 (52.7)	54 (21.1)	43 (16.8)	15 (5.9)	9 (3.5)
I feel in a good mood	36 (14.1)	82 (32.0)	97 (37.9)	33 (12.9)	8 (3.1)
I have frequent mood swings	66 (25.8)	73 (28.5)	47 (18.4)	41 (16.0)	29 (11.3)
I am unable to feel positive emotions	120 (46.9)	64 (25.0)	43 (16.8)	20 (7.8)	9 (3.5)
I feel angry/irritable	57 (22.3)	72 (28.1)	56 (21.9)	49 (19.1)	22 (8.6)
I have hunger attacks	80 (31.3)	77 (30.1)	31 (12.19)	39 (15.2)	29 (11.3)
I feel sad	43 (16.8)	76 (29.7)	65 (25.4)	54 (21.1)	18 (7.0)
I have the fear of gaining weight	66 (25.8)	46 (18.0)	39 (15.2)	51 (19.9)	54 (21.1)
I feel discouraged towards the future	31 (12.1)	71 (27.7)	54 (21.1)	52 (20.3)	48 (18.8)
I feel free from worries	149 (58.2)	61 (23.8)	38 (14.8)	6 (2.3)	2 (.8)
I feel abandoned	124 (48.4)	63 (24.6)	32 (12.5)	22 (8.6)	15 (5.9)
I feel full of initiative	75 (29.3)	81 (31.6)	71 (27.7)	19 (7.4)	10 (3.9)
I cannot understand clearly what emotion I am feeling	97 (37.9)	58 (22.7)	48 (18.8)	35 (13.7)	18 (7.0)
I feel unable to drive away unwanted thoughts, words, or ideas	97 (37.9)	57 (22.3)	49 (19.1)	34 (13.3)	19 (7.4)
I feel like I want to hurt myself/I hurt myself	178 (69.5)	37 (14.5)	19 (7.4)	16 (6.3)	6 (2.3)
I feel weak	94 (36.7)	76 (29.7)	38 (14.8)	36 (14.1)	12 (4.7)
I feel guilty if I eat too much	82 (32.0)	51 (19.9)	48 (18.8)	38 (14.8)	37 (14.5)
I feel assertive	75 (29.3)	92 (35.9)	56 (21.9)	22 (8.6)	11 (4.3)
I have suicide thoughts	192 (75.0)	41 (16.0)	13 (5.1)	3 (1.2)	7 (2.7)
I feel body discomfort	38 (14.8)	63 (24.6)	42 (16.4)	53 (20.7)	60 (23.4)
I easily cry	125 (48.8)	59 (23.0)	36 (14.1)	15 (5.9)	21 (8.2)
I feel trapped	89 (34.8)	61 (23.8)	40 (15.6)	27 (10.5)	39 (15.2)
I constantly think about diet	110 (43.0)	59 (23.0)	42 (16.4)	24 (9.4)	21 (8.2)
I feel frightened	93 (36.3)	80 (31.3)	43 (16.8)	23 (9.0)	17 (6.6)
I feel lonely	82 (32.0)	65 (25.4)	34 (13.3)	35 (13.7)	40 (15.6)
I have little appetite	104 (40.6)	60 (23.4)	44 (17.2)	30 (11.7)	18 (7.0)
I’m having a hard time getting things done	79 (30.9)	45 (17.6)	44 (17.2)	37 (14.5)	51 (19.9)
I can’t get interested in anything around me	108 (42.2)	65 (25.4)	42 (16.4)	23 (9.0)	18 (7.0)
I feel misunderstood	104 (40.6)	49 819.1)	37 (14.5)	36 (14.1)	30 (11.7)
I feel optimistic about the future	73 (28.5)	98 (38.3)	60 (23.4)	16 (6.3)	9 (3.5)
I have trouble sleeping and falling asleep	73 (28.5)	55 (21.5)	41 (16.0)	34 (13.3)	53 (20.7)
I feel a sense of emptiness	104 (40.6)	52 (20.3)	30 (11.7)	45 (17.6)	25 (9.8)
I need to repeat the same act as touching, counting, washing my hands	172 (67.2)	44 (17.2)	20 (7.8)	12 (4.7)	8 (3.1)
I feel guilty	154 (60.2)	48 (18.8)	21 (8.2)	23 (9.0)	10 (3.9)
I feel relaxed and calm	61 (23.8)	102 (39.8)	62 (24.2)	19 (7.4)	12 (4.7)
I have a tendency to abuse drugs	200 (78.1)	27 (10.5)	20 (7.8)	6 (2.3)	3 (1.2)
I have difficulty in memory and concentration	77 (30.01)	63 (24.6)	46 (18.0)	37 (14.5)	33 (12.9)

### Household and Psychological Status

The *t*-test was conducted (Levene’s test correction) using independent samples (Table 4) and psychological scales (i.e., Depression, Hostility, Anxiety, and Somatization and Positive Emotions), to verify if a difference exists in the psychological well-being of individuals who have experienced the lockdown with other people and those who underwent it alone.

**Table 4:** Household and psychological status (*N* = 256)

**Scale**	**Household with others**	**Household alone**	**Sign. two codes**	**t-Test after Levene’s correction**
**Hostility**	6.54 ± 4.1	3.9 ± 3.5	**.001**	3.493
**Depression**	18.8 ± 13.9	11.5 ± 10.9	**.001**	3.480
**Anxiety**	9.57 ± 6.5	5.38 ± 5.32	**.000**	4.144
**Somatization**	11.81 ± 6.6	7.58 ± 6.56	**.001**	3.439
**Positive Emotions**	6.87 ± 4.55	9.1 ± 4.8	.009	−2.652

The significant difference (*p* < .05) is indicated in bold

A significant difference was observed between the means (*p* < .05) of individuals living with others compared to those living alone. The means and standard deviations of people living with others indicated high scores. Therefore, individuals living with others were more depressed, anxious, and hostile and reported higher rates of somatization. Specifically, the Depression scale exhibited higher means (18.8 ± 13.9) compared with those of the other scales.

Additionally, the study conducted one-way ANOVA (Table 5) to explore differences between the psychological well-being of respondents (dependent variable) based on their household conditions (i.e., family, partners, and roommates) (independent variable).

**Table 5:** Household and psychological status (*N* = 256)

**Scale**	**Family**	**Partner**	**Roomates**				
	**(M ± sd)**	**(M ± sd)**	**(M ± s d)**	**df**	***F***	**Sign. two codes**	**Homogeneity of variances**
**Hostility**	**7.217 ± 4.15**	**4.55 ± 3.34**	5.6 ± 4.16	3	5.14	**.002**	.117
**Depression**	**21.16 ± 14.16**	**11.23 ± 10.41**	15.6 ± 12.51	3	5.9	**.001**	.080
**Anxiety**	**10.67 ± 6.49**	**6.0 ± 5.51**	7.35 ± 5.03	3	6.37	**.000**	.155
**Somatization**	12.5 ± 6.66	10.1 ± 6.27	9.35 ± 5.74	3	2.16	.093	.531
**Positive Emotions**	6.6 ± 4.57	7.0 ± 4.42	9.0 ± 4.25	3	1.44	.230	.776

The significant difference (*p* < .05) is indicated in bold

Levene’s test was conducted to examine the homogeneity of variance. Table 5 indicates that the variance was uniform for Hostility (*p* = .117), Depression (*p* = .080), Anxiety (*p* = .155), Somatization (*p* = .531), and Positive Emotions (*p* = .776).

A significant difference (*p* < .05) was observed between groups. Specifically, Bonferroni’s post hoc test pointed to significance (*p* < .05) between those living with family and partners and those living with family and roommates. The means and standard deviations of those living with family indicated higher scores in the Hostility, Depression, and Anxiety scales. Specifically, the Depression scale displayed higher means compared with those of the other scales. Thus, living with family during the COVID-19 pandemic led to significant self-reported difficulties in psychological well-being compared to those living with partners or roommates.

Moreover, MANCOVA was used to verify the relationship between household conditions, living alone or with others, and variables related to hormone therapy or lack of it (independent variables). Psychological scales (dependent variables) were used to explore whether undergoing hormone therapy influenced psychological well-being. Specifically, a significant association was noted for the Hostility, Depression, Anxiety, and Positive Emotions scales using both variables. Multivariate post hoc tests demonstrated that undergoing hormone therapy or not influences Depression (*p* = .005), Anxiety (*p* = .010), and Positive Emotions (*p* = .037), whereas household condition influences Hostility (*p* = .002), Anxiety (*p* = .000), and Depression (*p* = .001).

In the open-ended questions, the respondents added descriptions of experiences in their households during the pandemic, which revealed that living with family members during the COVID-19 pandemic led to an increase in episodes of discrimination, such as misgendering, body-shaming, and victimization from parents and relatives.

“I get misgendered by my dad every day, it happened even before but being able to see people who support me, made me feel better.”

“It’s a daily thing that before the lockdown could be avoided leaving the house, but now you are forced to suffer and endure, without seeing an end.”

### Access to Care and Services

Access to gender-affirming care was investigated by asking respondents whether the pandemic limited their search for prescriptions and if it contributed to the interruption of hormone therapy and influenced the administration of medicines. A total of 44.5% of the participants reported several fears regarding limited and denied access to medical care and specialized medical services for transgender/non-binary people (i.e., hormonal therapy, surgery, and legal intervention) due to the health emergency. In addition, 64.8% of the sample expressed concerns regarding failure to achieve their set goals and projects, such as undergoing surgeries, starting hormone therapy, and undertaking legal procedures to change identity documents.

#### Finding of Prescriptions and Medicines

The study explored the experiences of trans people regarding finding medicines and prescriptions during the health emergency and whether they needed to contact their doctor during the lockdown period. Analysis of the frequencies pointed out that 14.5% of the respondents experienced difficulties in finding prescriptions, whereas 9% had difficulties in finding medicines (see Table 6). Moreover, 18.8% of the entire sample reported having never consulted a specialist because they are not followed by any doctor.

**Table 6:** Access to medical devices

	***N* (%)**
***Difficulties in finding prescriptions (N* = *256)***	
Yes	37 (14.5)
No	217 (84.8)
Purchase medicines through unofficial ways	2 (.8)
***Difficulties in finding medicines (N* = *256)***	
Yes	23 (9)
No	177 (69.1)
No, because of sufficient supplies	56 (21.9)
***Need to contact the doctor (N* = *256)***	
Not followed by any doctor	48 (18.8)
Contacted the doctor for information regarding gender specialized services	44 (17.2)
Contacted the doctor to have information and prescriptions for therapy	122 (47.6)
Did not contact the doctor	88 (34.4)
***Interruption of hormone therapy (N* = *165)***	
Suspension because of lockdown restrictions	4 (1.6)
No suspension	161 (98.4)

#### Interruption of Hormone Therapy

The study further investigated whether respondents were forced to suspend hormone therapy because of the emergency. Table 6 indicates that only 4 out of the 165 subjects undergoing hormone therapy, suspended therapy due to lockdown restrictions.

#### Administration of Medicines

We investigated the difficulties encountered by the sample in the administration of intramuscular hormone therapies due to emergency restrictions. The results revealed that out of 99 individuals under intramuscular therapy, 6.6% had to contact a specialist for the administration of intramuscular therapy, 12.1% experienced quarantine with people who were able to help in the procedure, and 4.3% had to ask for help from people outside the family, such as friends or other relatives (Table 7).

**Table 7:** Difficulties in undertaking intramuscular therapy (*N* = 99)

	***N*** **(%)**
Had to contact a specialist outside the household	17 (6.6)
Had no difficulties doing the injection	23 (9.0)
Had no difficulties because quarantined with people who are able to do injections	31 (12.1)
Had to contact people outside of the household (relatives, friends) to do the injection	11 (4.3)
Had difficulties and had to provide alone	8 (3.1)
Had to ask to a family member	2 (0.8)

#### Access to Non-pharmacological Devices (i.e., Vaginal Dilators, Binders, Packers, Wigs, Make-Up, and Epilation Tools)

A total of 24.6% of the sample experienced difficulties due to the lockdown in terms of access to non-pharmacological devices, 39.5% experienced no difficulties, and 35.9% did not use such devices.

### Psychological Support

Nearly half of the sample (48.8%) reported undergoing psychological therapy, whereas 43.4% were not engaged in any psychological therapy. Regarding experiences related to psychological therapy during the lockdown, 5.9% of the sample did not receive an alternative to face-to-face therapy, whereas 9.8% of the respondents suspended therapy by choice of the therapist. Moreover, 25.4% of the sample reported receiving alternatives to face-to-face therapy, such as online therapy through Skype, Zoom, and other online platforms. The study also explored the experience of people that altered their therapy routine during the lockdown emergency and how they adapted to remote alternatives. The findings indicated that 4.7% of the respondents felt that the change was comfortable, whereas 4.4% mentioned that the situation led to privacy problems. Furthermore, a few subjects (2.3%) reported perceiving this change as invasive. Additionally, ANOVA was performed to explore any differences in the psychological well-being of respondents who started psychological therapy before the COVID-19 outbreak and continued it through the pandemic, those who started therapy before the pandemic but experienced interrupted support due to COVID-19, and those who did not receive any psychological support. Table 8 shows a significant difference (*p* < .05) in psychological well-being between groups. Bonferroni’s post hoc test pointed to a significant difference (*p* < .05) between those who started psychological care before COVID-19 and continued it during the pandemic, those who started therapy before the pandemic but had to suspend it due to lockdown restrictions, and those who were unengaged in any psychological therapy. The means and standard deviations of the respondents who started psychological therapy before the pandemic and continued it, and those who suspended it due to lockdown restrictions were higher on the Hostility scale. Moreover, the means of the respondents who started psychological therapy before the pandemic and continued it compared with those who did not undergo any kind of psychological therapy were higher in the Depression, Anxiety, and Somatization scales. Thus, the respondents who reported undergoing psychological therapy during the pandemic or suspended it due to the COVID-19 outbreak reported a lack of psychological well-being. Finally, Table 9 reveals that several subjects postponed therapy for personal reasons, such as economic and privacy concerns, or because they did not agree with the alternative method.

**Table 8:** Psychological well-being and psychological support (*N* = 256)

**Scale**	Psychological therapy continued during COVID-19^*^	Psychological therapy interrupted because of COVID-19^**^	**No psychological therapy**			
	**(M ± sd)**	**(M ± sd)**	**(M ± sd)**	**df**	***F***	**Sign. two codes**
**Hostility**	**7.184 ± 4.16**	**7.30 ± 4.25**	**4.88 ± 3.84**	2	10.36	**.000**
**Depression**	**21.36 ± 14.04**	18.65 ± 14.55	**13.71 ± 12.24**	2	9.72	**.000**
**Anxiety**	**10.84 ± 6.51**	9.65 ± 6.16	**6.84 ± 5.96**	2	12.126	**.000**
**Somatization**	**13.10 ± 6.80**	12.5 ± 6.42	**8.93 ± 6.20**	3	12.402	**.000**
**Positive Emotions**	6.6 ± 4.85	6.3 ± 3.93	7.96 ± 4.41	3	2.91	.056

^*^Respondents which started a psychological therapy before COVID-19 outbreak and continued it during the pandemic; ^**^Respondents which started a psychological therapy before COVID-19 but had to interrupt it because of the pandemic

The significant difference (*p* < .05) is indicated in bold

**Table 9:** Psychological therapy

	***N* (%)**
***Psychological support***	
Started before the COVID-19 emergency	125 (48.8)
Started immediately after the COVID-19 emergency	7 (2.8)
No psychological support	111 (43.4)
Interruption after the COVID-19 emergency	20 (7.8)
***Psychological support during the lockdown restrictions of COVID-19 emergency***	
No alternative to face to face therapy was proposed	15 (5.9)
An alternative to face to face therapy was proposed	65 (25.4)
Interruption for choice of the therapist	25 (9.8)
Acceptance to continue after initial refusal	4 (1.6)
No change because already by remote	4 (1.6)
***Reasons related to interruption of the patient after the COVID-19 emergency***	
Interruption for privacy reasons	3 (1.2)
Interruption for economic concerns	4 (1.6)
Interruption due to poor internet connection	1 (0.4)
Interruption for personal reasons	2 (0.8)
Feeling that it was not necessary	4 (1.6)
Interruption because an unwanted change was proposed	2 (4)
***Experience related to changes in therapy***	
Feeling comfortable	12 (4.7)
Privacy issues	11 (4.3)
No difference perceived	34 (13.3)
Feeling uncomfortable	6 (2.3)
Preference for face to face therapy	10 (3.1)
***Type of psychological support started after COVID-19***	
At the telephone	4 (1.6)
Online (Skype, Facetime, Zoom)	3 (1.2)
Used other methods	1 (0.4)

### Relationship with the Community

Descriptive analysis reveals that 48% of the participants reported a lack of positive, useful, and supportive initiatives in response to the emergency from the LGBT + and trans* organizations in Italy. In addition, the respondents reported a low perception of support initiatives at the local/regional/national levels (75.4%). The subjects stated, however, that online communication regarding their trans* identity during the emergency made them feel supported in 23% of cases.

## Discussion

The results indicate the impact of the COVID-19 pandemic on the transgender/non-binary population in Italy and provide information regarding the psychological status, access to medical health services, and factors that can represent relevant predictors of risk outcomes among transgender/non-binary people during lockdown restrictions.

The results show that the respondents experienced several difficulties during the COVID-19 pandemic in terms of mental, physical, and social well-being as well as certain difficulties related to access to health facilities. Among the respondents, the number of assigned females at birth was higher than assigned males at birth. This rate is consistent with international data that indicate a significant increase in transgender people assigned female at birth and transgender people assigned male at birth in recent years (Aydin et al., 2016).

Moreover, the results show a wide spectrum of gender identities that underline the tendency of transgender/non-binary people to maintain a more fluid and diversified identity as indicated by the latest research (Gosling, 2018; Richards et al., 2016).

In terms of psychological well-being, the respondents reported high levels of depression, hostility, anxiety, and discouragement about the future and described negative changes in psychological well-being between the period before the outbreak and the period throughout the emergency. These results are in line with those of previous studies (Bockting et al., 2016; Scandurra et al., 2017) and confirm that transgender individuals are significantly burdened by existing mental health issues. The results also suggest that the COVID-19 pandemic may have played a role in exacerbating such issues and added significant concerns for transgender individuals, which forced them to address different kinds of stressors. For instance, limited access to buffering features (e.g., social relationships and situations where one can express the self in the desired gender) was reported, which is in line with prior research (e.g., Trujillo et al., 2017), and played a significant role in the psychological well-being transgender individuals.

High levels of depression and anxiety were reported by individuals living with family members during the lockdown, which is considered an evidence of the impact of social distancing measures on the well-being of certain transgender people, exposing them to difficult situations.

In this regard, the open-ended questions highlighted that inside the households, transgender individuals experienced body-shaming, misgendering, and discrimination from parents and relatives, which indicates that households can represent a significantly unsafe environment for transgender individuals. Moreover, respondents undergoing psychological therapy throughout the pandemic reported high levels of depression, hostility, and anxiety and cited a lack of privacy in their households during psychological therapy.

This situation corroborates the negative impact that families may exert on the mental health of transgender individuals, which deprives them of protective buffering features (Klein & Golub, 2016). Notably, in terms of social support, the respondents pointed to the lack of initiatives from the LGBT + communities in response to the emergency. This aspect may have increased the sense of loneliness of transgender individuals in their households due to being unable to gain support from LGBT + communities. Indeed, previous research (Stanton et al., 2017) demonstrated that increased connectedness among LGBT communities represents a predictor of well-being. Another notable point is that the support promoted by such communities in Italy seems to be slightly present in contrast with international realities, where the literature (Hackimer & Proctor, 2015) states that transgender/non-binary people perceive active and beneficial support from LGBT + groups. Nevertheless, self-harming and suicide rates in the sample do not exceed 3%, which is in contrast to the review of Marshall et al. (2016), who demonstrated a high prevalence of non-suicidal self-injury and suicide. This result may suggest that the emergency and lockdown restrictions did not exert a severe impact on internalizing disorders. Thus, staying at home may have represented a comfortable environment for certain individuals according to one’s gender incongruence, which therefore resulted in a positive outcome.

The study observed a significant lack of family and social support and mental health issues. Thus, given the dearth of studies demonstrating the promotive effects of family support on the health of LGBT + subjects (Katz-Wise et al., 2016), the need to identify and intervene in situations of hostile households is imperative. In this respect, the need to increase the support on the territory among LGBT + communities and recommendation for the promotion of networks with specialized services and families to reduce stigma and violence are fundamental aspects. Importantly, the need to overcome the high prevalence rate of mental distress among transgender subjects by ensuring psychological support within gender-affirming care is imperative.

No critical aspects emerged regarding access to health care services. Only a small percentage of the sample felt the need to suspend or reduce hormone therapies, whereas difficulties were reported regarding access to medical supplies or prescriptions. Specifically, many individuals had issues in the administration of intramuscular hormones and had to ask for help from relatives or people outside the household. This result underlines the need for easily accessible specialized facilities to ensure therapeutic continuity. Additionally, a consistent percentage of the sample reported having never consulted any specialist, which is significant.

The data confirm the need to ensure the widespread distribution of specialized structures in Italy to avoid self-medication. Indeed, many transgender/non-binary subjects tend to purchase non-medical hormones online without prescription (Benotsch et al., 2013) to remedy the issues regarding specialized structures and the health system. A few examples are the long waiting lists and lack of networks between services, which render access to specialized structures difficult for those who live far away. Furthermore, significant concerns emerged regarding limited or denied access to specialized gender services and worries regarding future plans, such as delay in treatments or extension of waiting lists to undergo hormone therapies or reassignment interventions. The results indicate transgender, and gender-non-conforming people perceive gender-affirming care in Italy as lacking.

### Limitations

The findings should be interpreted with consideration of several limitations. The sample was collected exclusively online and implies a high socio-economic status. For this reason, it cannot be considered representative of the Italian transgender/non-binary population. Moreover, using online platforms to collect answers might have led to self-bias. Additionally, the mean age of the sample and the limited number of subjects undergoing hormone therapy provided a limited perception of difficulties related to obtaining medicines and prescriptions. Finally, the study would have benefited from a longitudinal design to obtain further evidence of the impact of COVID-19 on the transgender population over time. The study overlooked the analyses of covariates and the presence of a control group, which may provide a baseline for the assessment of the impact of COVID-19. Additionally, data on the subjects’ backgrounds are lacking; therefore, comparisons regarding their psychological well-being should be taken with caution.

However, the study was able to highlight critical issues and interesting aspects of the well-being of trans people during the COVID-19 pandemic.

## Conclusion

Despite its limitations, the study provided a framework for the condition of the transgender/non-binary population during the COVID-19 pandemic. The study findings suggested that the COVID-19 pandemic exhibited certain critical aspects related to transgender and non-binary people, such as psychological well-being within households, difficulty in accessing medicines and prescriptions, and the perceived lack of support from LGBT + communities in Italy. Such issues necessitate further considerations. Indeed, before the pandemic, many difficulties existed for transgender individuals, such as discrimination and misgendering within households, and access to health care, such as long waiting lists, poor networks among centers, and lack of cooperation among professionals. Nevertheless, the emergency, which limited social relationships, forced people to stay at home but overload health care systems, re-emphasized these critical aspects and stressed the need for transgender individuals to avoid such issues, which can negatively affect physical and psychological health. Therefore, the need to develop a bio-psychosocial and multifaceted perspective is imperative to improve access to care and network among services and develop a sense of community and involvement of families, especially for young trans people. In addition, the study underlined the need for institutions to implement integrated assessment and support that involve psychological and medical care to avoid the risk of self-medication and isolation.

## Supplementary Information

Below is the link to the electronic supplementary material.

Supplementary file1 (DOCX 46 KB)

## Data Availability

The manuscript has associated data in a data repository.
