# Supplementary material for: Psychological Well-Being of Trans* People in Italy During the COVID-19 Pandemic: Critical Issues and Personal Experiences
Source: Sex Res Social Policy. 2021 Aug 14;19(4):1808–18. doi: 10.1007/s13178-021-00633-3 (PMC8364416; doi:10.1007/s13178-021-00633-3)
Supplement: Supplementary file 1 — Supplementary file1 (DOCX 46 KB) [file 13178_2021_633_MOESM1_ESM.docx]

**COVID-19 and psychological well-being of trans* people in Italy Survey**

**SECTION I: Personal characteristics**

**1. Which pronoun do you usually use?**

**(You can give more than one answer)**

-He/him

-She/her

-You

-Them/theirs

- Others

**2. Which category/categories do you think best describes/describe your gender identity?**

**(You can give more than one answer)**

- Trans* person

- Male

- Female

- Non-binary person/gender queer/gender fluid

- Agender person

- No category

- Uncertain

- I am currently exploring my gender identity

3. **Gender of birth:**

- M

- F

- Other

Specify:

4. **How would you currently describe your gender identity?**

_______________________________________________

_______________________________________________

_______________________________________________

_____________________

**5. Would you like to add something about the meaning that this word/these words have for**

**you?**

_______________________________________________

_______________________________________________

_______________________________________________

_____________________

**6. Young people have very different identities. You can point from zero (not at all) to**

**ten (fully) how much you identify yourself with the following identities:**

**Male identity:**

Not at all__________________________________________________________Fully

**Female identity:**

Not at all__________________________________________________________Fully

**Non-binary:**

Not at all__________________________________________________________Fully

**7. About gender fluidity:**

• My gender identity is stable: it does not change over time

- Always

- Sometimes

- Never

• My gender identity is fluid: it changes in different contexts

- Always

- Sometimes

- Never

• My gender identity is fluid: it changes over time

- Always

- Sometimes

- Never

**8. Have you undertaken a social transition?**

- Yes

- No

• **How old were you when you started the social transition?** ______________________ **(Years and Months)**

• **Who is aware of your trans* identity*?**

- Everyone who knows me

- Only family and close friends

- Only a few people

- Other ___________________________________

• **In which contexts of your life are you currently living with the gender you identify with?**

- In all contexts

- In none

- Only in some contexts

- Other _________________________

• **If only some contexts, can you specify which ones?**

**____________________________________**

**9. Other considerations on my gender identity**:

_________________________________________________________________________________
_________________________________________________________________________________
______________________

**10. Age and date of birth:** __________________

**11. Education:**

- Elementary school

- Middle school

- High School Diploma

- Bachelor’s degree

- Masters’ degree

- Post graduate

**12. Profession:**

- Employee

- Freelance

- Student

- Unemployed

- Other _________________________

**13. Civil status:**

- Unmarried

- Married

- Separated

- Divorced

- Widowed

- Partner

**14. Current relationship situation:**

- Single

- In a relationship

- Other__________________________

**15. Do you have children?**

- No

- Yes

If yes, how many? _________

**SECTION II: Trans * identity and COVID-19**

**16. Before the decree of 11^th^ of March, who did you live with?**

- Alone

- With partner

- With parents/other relatives

- With roommates/flat mates

- Other____________________________________

**17. After the decree of 11^th^ of March, have you changed residence?**

-No

-Yes

-I considered it, but I did not move, or I could not do it

**18. Where do you currently live?**

-Center of a big city

-Suburbs of a big city

-Small or medium inland center

-Small or medium coastal town

-Isolated house

- Other_________________

**19. What type of accommodation do you currently live in?**

- Studio

- Two-room apartment

- Three-room apartment or more

**20. Does your home have a balcony or a garden?**

- Yes

- No

**21. Who do you live with?**

- Alone

- With others

If with others, please specify: ____________

**22. Did you continue to work?**

- Yes

- No

**23. Have there been changes in your work activity (e.g smart working, layoff)?**

- Yes

- No

- No, because I don’t work

**For many trans* people it can be difficult to find a place to work where they can feel comfortable and not suffer from discrimination. If you have lost your job or experienced changes in your employment status due to the current situation, do you want to add something?**

**___________________________________________________________________________________**

**_________________________________________________________________________**

**24. Are you currently facing financial difficulties due to the COVID-19 emergency?**

- No, I have no difficulties

- Yes, I have difficulties

- No, but I am concerned about the future

**25. Have you ever left the house since the COVID-19 emergency started?**

- Yes

- No

**26. If so, how many times a week on average?**

- 1

- 2

- 3

- 4

- More than 4

**27. When you left the house it was for**:

**(You can give more than one answer)**

- Shopping

- Going to the pharmacy

- Doing sport activities

- Have a walk

- Take care of a family member

- Take the dog out

- Other_____________________________________________________

**28. Are there people in your daily life who have been infected by COVID-19?**

-Yes

-No

**If yes, it is:**

**(You can give more than one answer)**

-Parent

-Partner

-Relative

-Close friends

-Acquaintance

-Colleague

-Other_________________

**29. Have you been in direct contact with this person/people?**

- Yes

- No

**30. Do you think you were infected by COVID-19?**

- Yes

- No

- I am not sure

**31. Have you been in mandatory quarantine because of direct contact with a patient with COVID-19?**

- Yes

- No

**32.Overall do you feel afraid of infection from COVID-19?**

- Yes, a lot

- Yes, quite

- Yes, partially

- No, I’m not afraid

- No, I don’t care

**33. Are you afraid that people you care about may have been infected/may be infected by COVID-19?**

- Yes, a lot

- Yes, quite

- Yes, partially

- No, I’m not afraid

- No, I don’t care

**34. How do you deal with the present emergency situation from COVID-19? (e.g. restrictive measures, social isolation...)?**

**(You can give more than one answer)**

- I strictly adhere to the rules imposed by the government, I consider them necessary

- I limit my movements leaving home only if necessary (shopping, going to the pharmacy, work, health reasons, assistance to a family member)

- I limit my movements, but sometimes I voluntarily meet other people even though I shouldn’t

- I consider the rules proposed by the government as excessive and unnecessary, but I respect them for

good of the community

- I consider the rules proposed by the government as excessive and unnecessary, I do not respect them

**35. Which of the following consequences have you experienced and/or lived or believe that you will experience/live because of COVID-19?**

**(You can give more than one answer)**

- Fear that projects or goals disappear

- Loss or decrease of work / living in a precarious condition

- Increase of violence in the family context, with partners (verbal, physical, sexual)

- Increase of discrimination because of my gender identity

- None or restricted access to food or health facilities

- None or limited access to general medical care and/or medical services for my trans* identity (e.g. hormonal therapy, surgical procedures, legal procedures...)

- None or limited access to services for psychological support of my trans* identity

(e.g. individual psychotherapy, group psychotherapy, counselling...)

- Other ________________________

**For some trans* people staying at home can be a source of stress and may expose the person to suffer an increase of abuse/discrimination within households. If it happened to you do you want to add something?**

**36. Did you avoid contacting a doctor to request a swab or did you avoid going to the hospital for the fear of being discriminated/abused?**

- Yes

- No

**37. If you requested access to services for your health, were you misgendered by the medical staff? (not recognized in the gender in which you identify yourself, called with incorrect names/pronouns)**

- Yes, always

- Yes, sometimes

- Yes, rarely

- No, never

**38. Have you suffered discrimination because of your gender identity when you requested**

**medical assistance/ did you go to an emergency room or to a general doctor/ have you been stopped for a normal check-up in the street?**

- Yes, always

- Yes, sometimes

- Yes, rarely

- No, never

**39. If it happened, do you want to tell us something more? (How did you feel, from whom you have suffered discrimination, what happened and how did you deal with the situation)**

**___________________________________________________________________________________**

**40. Did social isolation lead to an increase of discomfort regarding your gender identity?**

- Yes

- No

- If yes, why? _______________________________________________________________

**41. The condition of social isolation made you feel more comfortable with your gender identity? (e.g. have the perception of not undergoing acts of discrimination, not having the perception of having to comply with expectations of others...)**

- Yes

- No

- If yes, why:

____________________________________________________________

**42a. Regarding your trans identity*, have you observed positive, useful and supportive (e.g. initiatives, fundraising, live events) in response to COVID-19 emergency situation at an individual/community level?**

_____________________________

**42b. Regarding your trans identity*, have you observed positive, useful and supportive (e.g. initiatives, fundraising, live events) in response to COVID-19 emergency situation from LGBT+/trans* organizations?**

______________________________

**42c. Regarding your trans identity*, have you observed positive, useful and supportive (e.g. initiatives, fundraising, live events) in response to COVID-19 emergency situation at a local/regional/national level?**

______________________________

**SECTION III: Gender transition path**

**43. Are you currently undertaking a psychological/psychotherapeutic support?**

- Yes, I started it before the COVID-19 emergency began

- Yes, I started it after the beginning of the COVID-19 emergency **(go to question 49)**

- No **(go to question 51)**

- No, I suspended it after the decree of the 11^th^ of March **(go to question 46)**

**43a. If you started it before the COVID-19 how long has it been going on?**

**_______________**

**44. If you are currently undertaking a psychological/psychotherapeutic support who are you addressing to?**

- A private professional

- A gender identity service

- A clinic

- A hospital service

- Association/Non-profit organization

- Other**_______________________________**

**45. If you were offered an alternative way to continue the psychological/ psychotherapeutic support after the 11^th^ March decree, how did you respond?**

- I accepted immediately **(go to question 47)**

- I initially refused, but then I had a second thought and accepted **(go to question 47)**

- I did not agree and I interrupted

- Other ________________________________

**46. If you have voluntarily suspended, why did it happen?**

**(You can give more than one answer)**

- The suspension did not depend on me

- I suspended because I was proposed a method that I did not like

- I suspended for economic reasons

- I suspended because I do not have an adequate internet connection

- I suspended because I no longer felt the need for it at this time

- I suspended for privacy concerns

- I suspended because it felt invasive

- Other____________________________

**Are there other things you’d like to add regarding your psychological/psychotherapeutic support?**

**___________________________________________________________________________________**

**(continue with question 51)**

**47. If the psychological/psychotherapeutic support has continued after the decree of the 11^th^ of March, how are you proceeding with it?**

- Through online devices (Skype, Facetime, Zoom)

- By telephone

- In person

**48. If there has been a change how are you experiencing this change?**

**(You can give more than one answer)**

- I don’t feel any difference

- I feel it invasive

- I prefer it/I feel comfortable

- I have privacy issues

- There has been no change because I continue in person

-Other______________________

**Are there other things you’d like to add regarding your psychological/psychotherapeutic support?**

**___________________________________________________________________________________**

**(continue with question 51)**

**49. If you started it after the COVID-19 emergency, how are you proceeding with it?**

-Through online devices (Skype, Facetime, Zoom)

- By telephone

- In person

- Other__________________

**50. If you requested/started a psychological/psychotherapeutic support after the beginning of COVID-19 emergency, how did you find the professional/service you chose?**

**(You can give more than one answer)**

- I searched on the internet

- I received suggestions through Social Media (e.g. Facebook/Instagram ads)

- Through friends/relatives/acquaintances

- Through non-profit association/organization

- I already had a contact/s

- Other__________________________

**Are there other things you’d like to add regarding your psychological/psychotherapeutic support?**

**___________________________________________________________________________________**

**51. Are you currently undergoing a hormone therapy?**

- No, I have no intention to start a hormone therapy

- No, I haven’t started the hormone therapy yet

- Yes, and after the decree of the 11^th^ of March it was postponed

- Yes, between 0 and 3 months of hormone therapy

- Yes, between 3 and 6 months of hormone therapy

- Yes, between 6 and 12 months of hormone therapy

- Yes, between 12 and 24 months of hormone therapy

- Yes, I passed the 24 months of hormone therapy

- Other__________________

**52. Have you undergone any surgeries?**

- No, but I would like to undergo surgery

- No, I have no intention of undergoing surgery

- Yes, but I wasn’t on the waiting list/I didn’t have a specific date yet

- Yes, and after the decree of the 11^th^ of March it was postponed

- Other_____________________

**52a. If you want to undergo surgeries, indicate which ones:**

**___________________________________________**

**52b. If you undergone any surgery, please indicate the type of surgery you did:**

**(You can give more than one answer)**

-Breast and/or chest surgery

-Genital’s surgery

-Breast and/or chest and genital surgery

-Other__________________

**53. If your hormonal therapy/surgery authorization/surgical reassignment/legal change of** **registry/, was suspended due to the March 11^th^ decree, how did this make you feel?**

**(You can give more than one answer)**

- Angry

- Depressed

- Disappointed

- Uncertain about the future

- Frightened

- Calm

- Other___________________________

**Do you want to add anything?**

**___________________________________________________________________________________**

**54. Did you need to contact your endocrinologist?**

**(You can give more than one answer):**

- No

- I am not followed by any doctor

- Yes, to find a prescription for therapy

- Yes, to have information about the service

- Yes, to have information about the therapy

- Other________________________

**55. Did you need to suspend hormone therapy because of the restrictions imposed by the decree of the 11^th^ of March?**

**-**  No, because I didn’t start therapy

- No, I had enough stock

- No, I have unofficial ways to find medicines

- No, I borrowed medicines from my transgender friends

- Yes

- Other_________________________

**55a. If so, how did that make you feel?**

**(You can give more than one answer)**

- Angry

- Depressed

- Disappointed

- Uncertain about the future

- Frightened

- Calm

- Other___________________________

**56. Have you encountered difficulties in finding prescriptions because of restrictions imposed by the 11^th^ March decree?**

- No

- Yes

- I purchase of medicines through unofficial ways

**57. Have you encountered difficulties in finding medicines, because of restrictions imposed by 11^th^ March decree?**

- No

- Yes

- No, I borrowed medicines from my transgender friends

- No, I had enough stock

**58. Some medicines for hormone therapy are not prescribed through dematerialized prescriptions and, therefore, you cannot communicate to the pharmacist the NSE code.**

**Do you feel discriminated for that?**

- Yes

- No

- No, but I did not know

**59. Did you have to reduce the dosage yourself to make "economy" because of restrictions imposed by the decree of the 11^th^ of March?**

- Yes

- No, I had enough stock

- No, I have unofficial ways to find medicines

- No, I borrowed medicines from my transgender friends

**60. If you reduced/stopped hormone therapy, did it have negative effects on your psycho-physical well-being?**

- No

- Yes

- I don’t think so

- None of the previous

- Other_________________________________

**60a. If yes, indicate the type of symptoms perceived as a result of this change in dosage:**

**(You can give more than one answer)**

- Deep tiredness

- Depression

- Low strength

- Irritability

- Increase in sexual desire

- Decrease in sexual desire

- Feeling that the features obtained over time with hormone therapy

are decreasing

- Other__________________

**61. Are you currently undergoing intramuscular hormone therapy?**

- No, I do not use medicines administered intra-muscle

- Yes

**61a. If yes, how did you managed it before the 11^th^ March decree?**

- By myself

- With the help of a family member

- With a trusted person

- With a paid nurse

- Through a public service

**62. After the 11^th^ March decree how did you managed it?**

- I had no difficulty getting the injection because I usually do it by myself*

- I had no difficulty in doing the injection because I am in quarantine with people who are

able to make it

- I had to contact people outside the household to do it (professional nurse), but I managed to do it

- I had to contacts out of the household (relatives, friends), but I managed to do it

- I couldn’t find anyone willing to do it, so I "skipped" the vial

- I could not find anyone willing to do it, so I did it by myself* but not in the usual anatomical place (for example, thigh, arm, etc.)

- Other___________________________

**63. Has the current COVID-19 epidemic limited you in access/finding/use of non-pharmacological devices important for you (such as vaginal dilators, binders, packers, wigs, make-up, tools for epilation)?**

- Yes

- No

- I do not use any of them

**SECTION IV: Social and media**

**64. Before the COVID-19 emergency, on average, how much time you spent a day on social media (Instagram, Facebook) and messaging apps (WhatsApp, etc.)?**

- Never

- I only rarely access to social media

- Up to 30 minutes

- 30 to 60 minutes

- 1 to 2 hours

- 2 to 3 hours

- More than 4 hours per day

- All day long

**65. Currently, how much time do you spend on social media a day (Instagram, Facebook) and messaging apps (WhatsApp, etc.)?**

- Never

- I only rarely access to social media

- Up to 30 minutes

- 30 to 60 minutes

- 1 to 2 hours

- 2 to 3 hours

- More than 4 hours per day

**66. Currently, how much do you feel that virtual communication (social media, chat) can validly replace the personal one (face to face)?**

- A lot

- Fairly

- Little

- Not at all

**67. Currently do you have an adequate internet connection?**

- Yes

-No

**68. Currently do you have adequate digital devices (Pcs, tablets, smartphones etc.)?**

- Yes

- No

**69. How much spare time do you have these days?**

-More than usual

-Much more than usual

-Less than usual

-As usual

**70. How do you spend your free time these days?**

**(You can give more than one answer)**

- I listen to music

- Study

- Play a musical instrument

- Pray/I attend church

- Cook

- I carry out socio-political and voluntary activities

- Practice sports at home or outdoors

- I listen to and read the news on the web

- I read books

- Play video games

- I spend time on social media

- I spend my time on the telephone

- I spend my time surfing the web

- Household hobbies (model making, DIY, knitting...)

- I do something else

- I spend time with people who live with me

- I don’t do anything

**71. What kind of resources do you get information from?**

**(You can give more than one answer)**

- Social media

- Radio

- Trans Community*

- Friends/family

**72. Before the COVID-19 emergency, regarding your trans identity*, how much do you think that virtual communication and computer platforms make you feel supported?**

- A lot

- Fairly

- Partially

- Little

- Not at all

**73. Currently, regarding your trans identity*, how much do you think that virtual communication can make you feel supported?**

- A lot

- Fairly

- Partially

- Little

- Not at all

**74. Currently, how important do you think virtual closeness with the community trans*/LGBT+ is?**

- A lot

- Fairly

- Partially

- Little

- Not at all

**75. Which social media and/or communication/instant messaging tools do you use to get in touch with the community and you trans* friends?**

**(You can give more than one answer)**

- Facebook

- Instagram

- WhatsApp

- Skype

- Telegram

- Tumblr

- Zoom

- Googlemeet

- Houseparty

- Mobile phone calls

- Online dating applications (e.g. Tinder, Grindr)

- Other _____________________________

**SECTION V: Psychological well-being and COVID-19**

**Regarding the CURRENT SITUATION indicate the degree to which the following statements correspond to you: (From 0=not at all; 1=not at all; 2=moderately; 3=very 4=very much)**

76. I feel nervous

77. I feel anxious

78. I’m afraid of losing control

79. I don’t know what’s going on inside me

80. I feel in a good mood

81. I have frequent mood swings

82. I feel unable to feel positive emotions

83. I feel angry/irritable

84. I have sudden hunger attacks

85. I feel sad

86. I am afraid of getting fat

87. I feel uncertain/discouraged about the future

88. I feel free from worries

89. I feel abandoned

90. I feel full of initiative

91. I cannot clearly understand what emotion I’m feeling

92. I feel unable to drive out unwanted thoughts, words or ideas

93. I feel like I want to hurt myself/I hurt myself

94. I feel weak

95. I feel guilty if I eat too much

96. I feel energetic

97. I think about killing myself

98. I feel dissatisfied with my physical appearance

99. I easily cry

100. I feel like I’m trapped

101. I constantly think about diet

102. I feel scared

103. I feel lonely

104. I have poor appetite/increased appetite

105. I have trouble getting things done

106. I cannot feel interest in anything that surrounds me

107. I feel misunderstood

108. I feel optimistic about the future

109. I have difficulty sleeping and/or difficulty falling asleep

110. I feel a sense of emptiness

111. I need to repeat the same act as touching, counting, washing my hands

112. I feel guilty

113. I feel calm

114. I have a tendency to abuse medicines (alcohol, medicines)

115. I have memory and/or concentration difficulties

**116. With regard to these statements and emotional states, do you believe that there has been a**

**change between the period before the COVID-19 emergency and the current period?**

- No, I did not see any change

- There has been a positive change

- There has been a negative change

- I have seen some changes but I do not know whether positive or negative

- Other__________________________

**Specify:**

**Have you encountered any difficulties that have not been mentioned in this survey that you would like to add?**

____________________________________________________________________________________

____________________________________________________________________________________

____________________________________________________________________________________

**Are there some suggestions you’d like to give to the people who belong to trans*/LGBT+ communities in this time of great difficulty?**

____________________________________________________________________________________

____________________________________________________________________________________

____________________________________________________________________________________

Thank you for your contribution.

We thank you very much for your time spent on this research.

If you are interested* to participate to an interview on the topics of this research, please insert here your email:
